# The Role of Serine Proteases and Antiproteases in the Cystic Fibrosis Lung

**DOI:** 10.1155/2015/293053

**Published:** 2015-06-21

**Authors:** Matthew S. Twigg, Simon Brockbank, Philip Lowry, S. Peter FitzGerald, Clifford Taggart, Sinéad Weldon

**Affiliations:** ^1^Centre for Infection and Immunity, School of Medicine, Dentistry and Biomedical Sciences, Queen's University Belfast, Health Sciences Building, 97 Lisburn Road, Belfast BT9 7AE, UK; ^2^Randox Laboratories Limited, 55 Diamond Road, Crumlin, County Antrim BT29 4QY, UK

## Abstract

Cystic fibrosis (CF) lung disease is an inherited condition with an incidence rate of approximately 1 in 2500 new born babies. CF is characterized as chronic infection of the lung which leads to inflammation of the airway. Sputum from CF patients contains elevated levels of neutrophils and subsequently elevated levels of neutrophil serine proteases. In a healthy individual these proteases aid in the phagocytic process by degrading microbial peptides and are kept in homeostatic balance by cognate antiproteases. Due to the heavy neutrophil burden associated with CF the high concentration of neutrophil derived proteases overwhelms cognate antiproteases. The general effects of this protease/antiprotease imbalance are impaired mucus clearance, increased and self-perpetuating inflammation, and impaired immune responses and tissue. To restore this balance antiproteases have been suggested as potential therapeutics or therapeutic targets. As such a number of both endogenous and synthetic antiproteases have been trialed with mixed success as therapeutics for CF lung disease.

## 1. Introduction to Cystic Fibrosis

Cystic fibrosis (CF) is an autosomal recessive genetic disorder caused by loss of expression or functional mutations to the cystic fibrosis transmembrane conductance regulator (CFTR) [1, 2]. CF affects multiple organs; however the majority of the pathology related to CF is due to its effect on the respiratory system. Nonfunctional CFTR channels in CF patients prevent the regulation of chloride and sodium ions across epithelial membranes leading to increased and dehydrated mucus secretions in the lungs [1]. CF patients have an impaired ability to clear this mucus due to damage caused to the cilia structures in the lungs and as such are therefore highly susceptible to chronic bacterial infections within the lung which are effectually impossible to eradicate [3]. The ramification of chronic bacterial infection is a sustained and detrimental inflammatory response from the body's innate immune system [4, 5]. There are many factors which mediate the inflammatory response to chronic bacterial infection in CF; these include proinflammatory cytokines such as IL-1*β*, IL-6, IL-8, GM-CSF, and TNF-*α* [6, 7].

**Figure 1:**
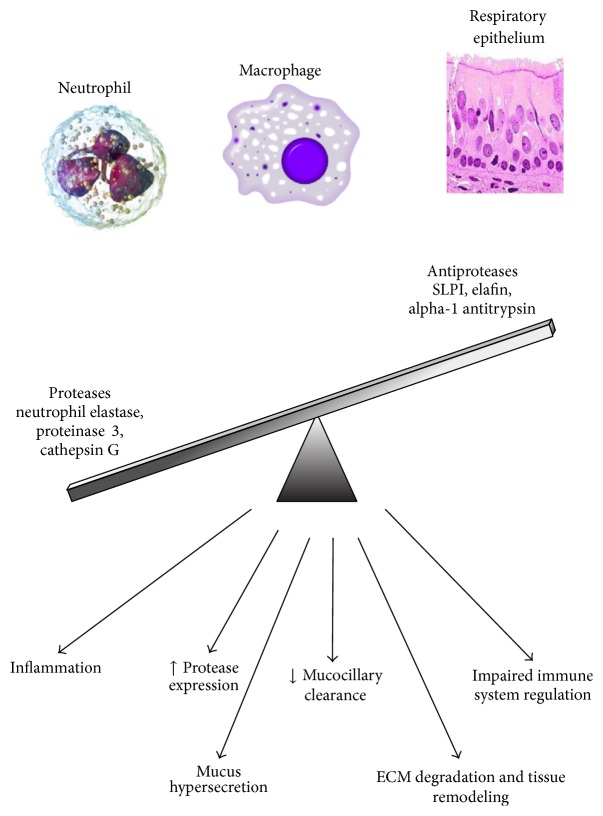
In the cystic fibrosis lung, antiprotease production by both innate immune cells and respiratory epithelial cells is overwhelmed by protease production resulting mainly from neutrophils. This leads to a disruption of the homeostatic protease/antiprotease balance resulting in a number of detrimental effects causing increase lung pathology.

One of the key immune cell mediators of this detrimental inflammatory response seen in CF patients is polymorphonuclear neutrophils [8]. In CF lungs, neutrophils represent ~70% of the inflammatory cell population in contrast to ~1% in epithelial lining fluid from healthy lungs [9]. Neutrophils are recruited to these sites of infection by increased expression of chemoattractants such as IL-8 by lung epithelial tissue [10]. Once recruited, neutrophils are activated and release a wide variety of molecules, such as proteases, DNA, and reactive oxygen species in an attempt to combat bacterial infection, further driving the inflammatory response and causing progressive tissue damage [8]. Evidence to date supports the hypothesis that CF neutrophils may be inherently defective [8, 11–13]. In addition to the release of proinflammatory mediators, neutrophils in the CF lung are not successfully cleared via macrophage phagocytosis [5, 14]. Neutrophil necrosis further increases the levels of proinflammatory mediators, increasing tissue damage and also increasing the viscosity of the CF patients sputum [14]. In healthy individuals tissue damage as a result of inflammation is in part controlled by homeostatic regulation of proteases via antiprotease activity. Inflammation observed in CF patients mainly as a result of neutrophil activity is highly disruptive to this protease/antiprotease balance as illustrated in Figure 1. The role that these serine proteases and their inhibitors play in the CF lung in either protecting the lung tissue or contributing to pathology will be the subject of this review.

## 2. Neutrophil Serine Protease Activity in CF

Proteases degrade proteins into either polypeptides or amino acids and are grouped on the basis of their catalytic residues. The 4 groups of proteases are serine proteases, cysteine proteases, metalloproteases, and the less common aspartic acid proteases [15, 16]. Neutrophil serine proteases are the main proteases implicated in the damage observed in the lungs of CF patients; these are neutrophil elastase (NE), proteinase 3 (PR3), and cathepsin G (Cat G) [17]. All three are members of the chymotrypsin family, and are expressed by neutrophils [17]. Upon translation these proteases appear as inactive precursor peptides referred to as zymogens. All three serine proteases undergo a two-stage posttranslational modification process in order to produce their active mature forms. The initial stage is the cleavage of an N-terminal signal peptide by a signal peptidase. The second stage is the cleavage of a prodipeptide from the N-terminal by the cysteine protease cathepsin C, which is required for enzymatic activity, and the cleavage of a C-terminal propeptide which may be required for packaging of the mature protein [18–22].

The mature forms of NE, PR3, and Cat G are stored in azurophilic granules within the cytoplasm of neutrophils. The activities of all three of these proteases are reliant on an amino acid triad composed of aspartate, histidine, and serine residues [18]. These residues are interspersed at different positions in the primary structure of each of the three serine proteases; however these residues are brought together in an active site region in the tertiary structure [15]. Serine proteases act either intracellularly, degrading microbial proteins in the phagosome, or extracellularly, regulating the immune system and aiding the degradation of extracellular matrix (ECM) components [18]. Owing to their broad range activity, the lack of regulation of these proteases in the CF lung is highly detrimental. The majority of research into the role of neutrophil serine proteases in the lung has focused on NE; however PR3 and Cat G are found at high concentrations in the sputum and bronchial alveolar lavage fluid (BALF) of CF patients so they therefore should not be discounted [23, 24].

### 2.1. Neutrophil Elastase

NE is a 29 kDa serine protease expressed by neutrophils from the gene* ELANE*, located on chromosome 19 [25]. NE is secreted upon neutrophil activation, into the phagosome during phagocytosis or released during neutrophil necrosis. Due to the heavy neutrophil burden associated with CF discussed previously, the levels of NE in the CF airway have been shown to reach micromolar concentrations [26]. Increased levels of NE in the CF lung have been attributed to elevated neutrophil numbers; however, defective neutrophil degranulation may also play a role [27, 28]. CF neutrophils were shown to release greater levels of elastase than non-CF controls despite the fact that the total complement of NE in the CF neutrophil was similar between groups [28]. This increased release may be attributable in part to the inflammatory milieu in the CF lung or as a result of CFTR mutation and/or dysfunction in the cell [27–29]. Further work is needed to fully understand the mechanisms of elevated NE activity in CF. In a healthy individual NE functions to cleave microbial peptides liberated during phagocytosis [30]. However in CF, the elevated level of NE overwhelms the host's cognate regulation of this protease and as such has profound detrimental effects. The general effect of increased NE levels in the CF lung can be grouped into the following categories: impaired mucociliary clearance, airway remodeling, proinflammatory activity, and the impairing of both the innate and adaptive immune system. The impairment of mucociliary clearance mainly revolves around the interactions between NE and mucins. Mucins are a family of highly glycosylated proteins produced by epithelial cells and are the main components of the mucus found clogging the airways of CF patients [31, 32]. NE has been shown to regulate the mucins MUC5AC and MUC2 via activation of TNF*α*-converting enzyme which upregulates the expression of these mucins via the epidermal growth factor receptor (EGFR) pathway [33–35]. The mucins MUC4 and MUC1 are also upregulated by NE; however their function in the lung is less understood [36–38]. NE has also been shown to cause the hypersecretion of both MUC5AC and MUC5B via the activation of protein kinase pathways further increasing mucus production and its secretion into the CF airway [39, 40]. Finally NE has the ability to reduce ciliary beat frequency and degrade cilia structures. The combined effect of reducing ciliary beat frequency and degradation of cilia prevents mucus from being removed from the airways therefore increasing mucus plugging in CF patients, providing potential colonization sites for bacteria [41–43].

When at high concentrations as found in the CF lung, NE causes airway remodeling owing to the degradation of ECM proteins in the airway such as elastin and fibronectin [44]. The disruption of cell surface structures by NE aggravates neutrophil mediated inflammation increasing expression of the proinflammatory cytokine IL-8 by airway epithelial tissue [28, 45–47]. This increase in IL-8 may be mediated via NE activation of the TLR-4 or EGFR cell signaling pathways, or through the TLR-2 pathway due to the cleavage of CXCR1 receptors from neutrophil cell surfaces [46, 48, 49]. NE release from neutrophils in the lung induces IL-8 expression leading to further neutrophil recruitment resulting in a self-perpetuating and detrimental cycle of neutrophil mediated inflammation. In addition to having proinflammatory activity via acting upon IL-8 levels, NE has also been shown to directly upregulate the proinflammatory matrix metalloproteases (MMPs): MMP-9 and MMP-4 [50]. Indirect activation of MMP-9 can also be mediated by NE due to its ability to inactivate the cognate inhibitor of MMP-9: TIMP-1 [51].

Excess NE levels in the CF lung may negatively affect both the innate and adaptive immune systems. NE both cleaves and downregulates flagella, an important bacterial pathogen-associated molecular pattern (PAMP) [52, 53]. Flagella cleavage has the effect of reducing the innate immune system's ability to detect pathogens such as* Pseudomonas aeruginosa* via TLR signaling pathways [54]. Detection and clearance of pathogens is also inhibited by excess NE due to cleavage of opsonizing peptides C3bi, CR1, and C5 receptor site, rendering reduced phagocytic ability [55, 56]. NE has also been shown to degrade antimicrobial peptides such as lactoferrin and *β*-defensins directly inhibiting bacterial killing [57, 58]. Finally NE has been shown to reduce the ability of macrophages to clear apoptotic cells due to its ability to cleave macrophage apoptotic cell receptors such as CD36 [59]. In addition to inhibition of the innate immune system, NE inhibits the adaptive immune system as research has shown that NE cleaves T cell receptors CD2, CD4, CD8, and CD14, impairing monocyte activation and also blocking dendritic cell maturation and antigen presentation [60, 61]. The combined detrimental effects of excess NE in the CF lung may result in increased bacterial survival rates heavily contributing to the state of chronic infection associated with CF.

NE can be found associated with the DNA structures secreted from activated neutrophils called neutrophil extracellular traps (NETs). NETs are known to be produced as a result of reactive oxygen species; however downstream of this, NE has been shown to regulate the formation of NETs, with studies showing that NE knockout mice have an inability to form NETs in a* Klebsiella pneumonia* infection model [62, 63]. The translocation of NE to the nucleus upon neutrophil activation and its subsequent degradation of specific histones promotes chromatin decondensation; this process has been shown to be further driven by another enzyme associated with neutrophil granulocytes: myeloperoxidase [63, 64]. The role of NE directly associated with NETs in the CF lung is however poorly understood and currently under investigation. Due to the high neutrophil burden present in the CF lung these NET structures account for a significant proportion of the DNA content found in mucus [65]. The presence of this extracellular DNA in mucus increases its plasticity increasing mucus plugging [66]. NE associated with the NET structures has been shown to be less active when it is associated with DNA; however it is also significantly less susceptible to the actions of cognate and therapeutic protease inhibitors [67]. It is therefore speculated that NET associated NE could act as a reservoir for NE in the CF lung [67]. DNase treatment of sputum has been shown to significantly increase the activity of NET associated NE but also render it susceptible to inhibition to cognate protease inhibitors. A combination of DNase treatment and protease inhibitor may be a potential therapeutic treatment to alleviate the detrimental inflammatory burden of NE in CF patients [67, 68]. Due to the wide variety of detrimental effects associated with high levels of NE found in CF patients, NE is regarded as the main protease responsible for inflammatory tissue damage in the CF lung.

### 2.2. Proteinase 3

PR3 is a 29 kDa, 222-amino acid serine protease expressed from the* PRTN3 *gene by activated neutrophils [69, 70]. The biological role of PR3 in degrading microbial peptides is similar to that of NE; however, PR3 has been shown to degrade IL-8 resulting in a truncated form of the chemokine with more potent neutrophil chemoattraction activity [71]. This increase in chemoattraction has the potential to further potentiate the detrimental cycle of inflammation previously described for NE. PR3 has also been shown to increase the interaction between neutrophils and the IL-8 receptor CXCR1 [71]. The increases in both neutrophil recruitment and receptor interaction with IL-8 caused by PR3 have clear detrimental implications for the cycle of neutrophil derived inflammation which results in tissue damage within the CF lung.

### 2.3. Cathepsin G

The remaining serine protease produced by neutrophils implicated in CF lung disease is Cat G. Although Cat G is a serine protease, it belongs to a larger family of proteases which encompass the cysteine cathepsins B, C, H, L, S, K, O, F, X, V, and W and aspartic acid proteases (cathepsins D and E) [72]. Mature Cat G is 28.5 kDa in size and composed of 235 amino acid residues [73]. Cat G is released upon neutrophil activation and possesses the ability to degrade structural components of the extracellular matrix when at high concentrations [17]. Cat G will inhibit the actions of macrophages in clearing apoptotic cells from CF airways; this leads to a rise in neutrophil necrosis and therefore the uncontrolled release of proteases into the lung [17]. Finally Cat G in CF BALF has been shown to have the highest potency of all three neutrophil serine proteases to degrade surfactant protein A, a peptide that facilities microbial clearance by macrophages, the result of which is a reduction in macrophage phagocytic activity, and therefore increased bacterial survival [74].

## 3. Antiprotease Activity in CF

In a healthy lung, antiproteases maintain a homeostatic balance with proteases, preventing the associated inflammatory damage which results from excess protease activity. In the CF lung it is the inability of these antiproteases to regulate their cognate proteases that is in part responsible for the pathology associated with infection and inflammation. A number of antiproteases associated with CF are discussed below.

### 3.1. Introduction to the WFDC Protein Family

The whey acidic protein (WAP) four disulphide core (WFDC) proteins are a family of putative multifunctional host defense proteins. These proteins possess a WAP domain composed of approximately 50 amino acid residues with eight conserved cysteine residues which form four disulphide linkages [75]. To date there have been 18 WFDC proteins identified and, in humans, the majority of these proteins are transcribed from genes located on chromosome 20. However two WFDC proteins have been found to be transcribed from genes of chromosome 17 [76, 77]. The best characterized members of this family are secretory leukocyte protease inhibitor (SLPI) and elafin, both heavily implicated in the protease/antiprotease balance in the lung.

### 3.2. SLPI

SLPI is an 11.7 kDa cationic serine protease inhibitor which forms a constituent part of the body's antiprotease screen [78]. Following posttranslational processing, the mature SLPI peptide consists of 107 amino acid residues and possesses two WFDC domains, each of which has four disulphide linkages [75]. SLPI has been detected in a variety of patient samples and is produced by a number of cell types including neutrophils, macrophages, serous cells of bronchial submucosal glands and nonciliated bronchial epithelial cells [79–81]. SLPI is expressed in response to various stimuli such as bacterial lipopolysaccharides, NE, and a number of cytokines [80–84]. SLPI exerts antiprotease activity against NE, cat G, trypsin and chymotrypsin, mediated via its C-terminal WFDC domain [85]. The key active site amino acid residue in SLPI is Leu^72^ since point mutation of this residue abolishes the antiprotease activity of SLPI [85].

In addition to protease inhibition SLPI has also been shown to inhibit inflammatory responses via a number of differing mechanisms, both extracellularly and intracellularly. SLPI acts extracellularly directly binding bacterial lipopolysaccharide (LPS) and lipoteichoic acid (LTA), preventing TLR activation [86]. SLPI acts intracellularly by preventing the LPS/LTA induced activation of NF-*κ*B, competing with p65 for binding to NF-*κ*B sites in the promoter regions of pro-inflammatory genes such as IL-8 and TNF*α* therefore preventing the expression of these proinflammatory cytokines and inhibiting the degradation of I*κ*B*α* and IRAK which in turn prevents NF-*κ*B activation [86–88]. The antiprotease and anti-inflammatory functions of SLPI can however be disrupted due to cleavage by excess concentration of its cognate substrates. SLPI cleavage by the cysteine proteases cathepsins B, L, and S between residues Thr^67^ and Tyr^68^ disrupts the active site of SLPI abolishing its ability to inhibit NE [89]. Further work demonstrated that SLPI levels are reduced in CF patients infected by the opportunistic pathogen* P. aeruginosa* [90]. Western blot analysis of BALF from these patients showed SLPI to be cleaved, an observation not seen in CF patients who were negative for* P. aeruginosa* [90]. Further investigation identified this cleavage of SLPI to be caused by excessive levels of NE present as a result of* P. aeruginosa *infection [90]. NE-cleaved SLPI loses the ability to bind LPS and NF-*κ*B consensus oligonucleotides [90]. NE-cleaved SLPI however maintains some antiprotease activity as this activity is mediated by the C-terminal domain which remains intact after cleavage [90]. The proteolytic cleavage of SLPI by NE could have implications for its use as an anti-inflammatory therapeutic in CF patients.

### 3.3. Elafin

Elafin, like SLPI, has been shown to be expressed in macrophages and neutrophils [77]. Mature elafin is formed from the cleavage of a 12 kDa, 117-amino acid long precursor peptide called preelafin or trappin-2 by tryptase [91, 92]. The mature elafin peptide has a relative mass of 6 kDa and possesses 57 amino acid residues [91, 93]. Elafin has two conserved domains, a cementoin domain which acts as a substrate for the enzyme transglutaminase, mediating the incorporation of elafin into extracellular matrix proteins, and the characteristic WFDC domain [76, 94]. The high level of homology seen in the WFDC domain between SLPI and elafin would suggest that like SLPI, elafin functions as a protease inhibitor. This was shown to be the case as elafin, like SLPI, possesses antiprotease activity against NE, trypsin, and chymotrypsin [93, 95]. However, unlike SLPI elafin possesses antiprotease activity against PR3 but not against Cat G [93].

In addition to antiprotease activity, elafin also possesses anti-inflammatory activity, and similar to SLPI, functions in both an intracellular and extracellular manner. Elafin has been shown to inhibit NF-*κ*B activation in monocytes stimulated by LPS and LTA therefore causing a reduction in inflammatory cytokine expression [96]. Elafin was also shown to inhibit proteosome pathways evidenced by the buildup of ubiquitinated IRAK-1 and I*κ*B*α* in LPS-stimulated monocytic cells [96]. Elafin, like SLPI, will also neutralize LPS.* In vivo* recombinant trappin-2 has been shown to reduce proinflammatory cytokines MIP-2, KC (murine IL-8 homologue), and TNF-*α* in LPS-treated mice [97]. Neutrophil influx into the lung and protease activity was also seen to be reduced in mice treated with recombinant trappin-2 and stimulated with LPS [97, 98]. Elafin has been proposed as a therapeutic in the treatment of pulmonary arterial hypertension with Proteo Biotec Inc. carrying out phase I clinical trials of elafin with measured success. Elafin treatment for pulmonary arterial hypertension is currently in phase II trials. If the trialling of elafin to treat pulmonary arterial hypertension is successful, it could be used as a potential treatment in CF. Experimental evidence has however shown that like SLPI, elafin is proteolytically cleaved by excess NE in the BAL fluid of CF patient infected with* P. aeruginosa* [99, 100]. The effects of this cleavage were the inactivation of elafin's anti-neutrophil elastase activity due to cleavage of the protease-binding loop. Interestingly the antibacterial properties of elafin were not affected by cleavage [100]. Proteolytic cleavage of elafin may have implications for its efficacy when being used as a therapeutic* in vivo*. Recent work has shown that mutating key residues at the NE cleavage site in elafin results in a peptide with similar antiprotease activity as the wild-type form but with significantly increased stability and anti-inflammatory activity [101]. When compared to wild-type, the mutant forms of elafin were shown to have improved LPS neutralizing activity* in vitro* and increased anti-inflammatory activity when employed in an acute model of pulmonary inflammation induced by* P. aeruginosa *LPS [101].

In addition to their antiprotease and anti-inflammatory function both SLPI and elafin have been shown to possess antimicrobial activity against both Gram negative and Gram positive organisms [102]. Both SLPI and elafin are cationic peptides and this property may mediate their antibacterial activity. Bacterial species reported to be susceptible to SLPI and elafin include* P. aeruginosa* and* Staphylococcus aureus* both of which are heavily implicated in the colonization of adult and juvenile CF patients, respectively [102, 103]. Although SLPI's antibacterial activity is mainly mediated by the N-terminus of the peptide, antibacterial activity was observed to be maximal when the peptide was complete with mutation of the C-terminal WFDC domain shown to result in a slight reduction in antibacterial function [102]. The antibacterial activity of elafin is mediated by both the WFDC domain and the cementoin domain [104]. Interestingly the precursor peptide to elafin, trappin-2, has been shown to possess greater antibacterial activity than the mature peptide [103].

### 3.4. Alpha-1 Antitrypsin

Alpha-1 antitrypsin (AAT) is a serine protease inhibitor shown to be active against trypsin, plasmin, Cat G, MMP-12 in addition to NE [105–108]. AAT is expressed as a 418-amino acid protein which undergoes posttranslational cleavage of a 24-amino acid signal peptide and glycosylation to form a 52 kDa mature peptide [109]. Expression of AAT mainly takes place in hepatocytes; however, AAT expression has been observed in respiratory epithelial cells, macrophages, and neutrophils [109, 110]. Aerosolized AAT therapy has been proposed as a treatment for the inflammatory damage caused by neutrophil serine proteases in CF patients for a number of years. However a trial conducted with aerosolized AAT showed only a tending relationship toward reduced NE in patients treated with AAT after 4 weeks and did not identify any anti-inflammatory effects of AAT treatment [111]; this trial did however show significant reductions in NE/AAT complexes and myeloperoxidase in the AAT treated cohort [111]. Furthermore, CF patients receiving aerosolized AAT did not exhibit significant reductions in* Pseudomonas* counts in comparison to the placebo group [111]. The results of the Martin et al. [111] study were however contradicted by a second clinical trial published one year later which showed AAT to have significant anti-inflammatory effects [112]. This trial involved the aerosolized delivery of AAT to CF patients and found a significant reduction in NE, IL-8, IL-1*β*, and TNF-*α* levels after 4 weeks of treatment in comparison to baseline; however no increase in FEV1 was observed [112]. In addition, AAT treatment reduced neutrophil counts and there was a reduction in* P. aeruginosa* CFUs [112]. The ability of AAT to regulate its cognate proteases in the CF lung may however be curtailed by high reactive oxygen species levels as a result of increased neutrophil burden [112, 113]. Oxidation of residue Met358 in the active site of AAT renders the protease inactive [47]. AAT can also be cleaved and inactivated by MMPs [114, 115]. If MMP cleavage of AAT takes place in the CF lung the effects could potentially be decreased inhibition of neutrophil serine proteases and therefore continuing the detrimental effects of these peptides. Martin et al. [111] and Griese et al. [112] are the two most recent clinical trials using ATT in CF patients; however there are a number of NIH funded trials currently in progress in the US investigating the use of ATT in CF treatment [111, 112]. The results of these trials may provide further clarification on the efficacy of using this antiprotease as a CF therapeutic.

### 3.5. Synthetic Serine Protease Inhibitors

In addition to the endogenous protease inhibitors such as SLPI and elafin being trialled for use as anti-inflammatory therapeutics in CF, a number of synthetic inhibitors of NE have been developed and trialed. NE was chosen as a target for inhibition by synthetic compounds due to its being widely recognized as the key serine protease connected to lung pathology in CF. DX-890 is a small protein inhibitor of NE, which has been shown to be tolerable in rat and in humans after a phase I clinical trial [116, 117]. This compound was shown to inhibit NE released from both healthy and CF neutrophils when treated at concentrations above 100 nM [118]. DX-890 also reduced IL-8 release from both healthy and CF neutrophils and reduced neutrophil transmigration through the epithelial barrier [118]. A second trial using DX-890 as a NE inhibitor in CF showed it to be only partially effective at inhibiting NE in CF sputum; however this study only tested DX-890 against low molecular ratios of NE, which do not adequately represent the levels observed in the CF lung [119]. Another compound trialled is AZD9668, an orally administered reversible inhibitor of NE [120]. A phase II clinical trial looking at its efficacy in bronchiectasis showed reductions in proinflammatory cytokines IL-6 and IL-8 and improvements in FEV_1_; however sputum neutrophils were not shown to be decreased [121, 122]. A trial looking at AZD9668 use as a CF therapeutic showed similar results, with reports of a reduction in pro-inflammatory cytokines which was postulated to be due to inhibition of elastin cleavage by NE [123].

## 4. Conclusion

An increasing volume of experimental evidence points to the importance of proteases and their cognate protease inhibitors in CF lung disease. The imbalance of the protease/antiprotease balance in favor of the neutrophil serine proteases results in a self-perpetuating cycle of inflammation and respiratory tissue damage. This evidence also points to therapeutic options for the treatment of CF patients to reduce the inflammatory tissue damage in the form of antiprotease therapy using either synthetic antiproteases or mutated endogenous antiproteases. Clinical trials have shown that the application of antiproteases such as SLPI and AAT results in the reduction of inflammation due to the restoration of the protease/antiprotease balance. The therapeutic and diagnostic applications of research into proteases and antiproteases in the CF lung continue to attract significant interest.
